# AI‐Assisted Literature Mining Reveals Spatiotemporal Heterogeneity and Progression Trajectories of Traditional Chinese Medicine Syndromes in Coronary Heart Disease in China

**DOI:** 10.1111/jebm.70162

**Published:** 2026-07-07

**Authors:** Qianzi Che, Tian Song, Zhaoyuan Gong, Chuxuan Wang, Bin Liu, Mingzhi Hu, Ning Liang, Haili Zhang, Huizhen Li, Qingyang Zeng, Lin Chen, Yuanming Leng, Xihao Cao, Jing Wan, Jing Guo, Nannan Shi

**Affiliations:** ^1^ Institute of Basic Research in Clinical Medicine China Academy of Chinese Medical Sciences Beijing China; ^2^ School of Traditional Chinese Medicine Beijing University of Chinese Medicine Beijing China; ^3^ Computer Department Beijing University of Chemical Technology Beijing China; ^4^ Biostatistics Department, School of Public Health Boston University Boston USA

**Keywords:** Chinese medicine syndrome, coronary heart disease, sequential pattern mining, spatiotemporal analysis, traditional Chinese medicine

## Abstract

Despite the centrality of syndrome differentiation in guiding personalized traditional Chinese medicine (TCM) interventions for coronary heart disease (CHD), existing studies of TCM syndrome distribution are constrained by fragmented methodologies and limited spatiotemporal resolution. In this study, we employed an artificial intelligence (AI)‐assisted literature mining framework combined with statistical and pattern‐mining analyses to characterize the spatiotemporal patterns and evolutionary dynamics of CHD syndrome elements across China. We mined data from 651 peer‐reviewed publications reporting 234,656 CHD patients, published between January 2000 and December 2023. A customized constrained Sequential PAttern Discovery using Equivalence classes algorithm was applied to quantify syndrome transition trajectories, while hierarchical clustering integrated with similarity‐matrix heatmaps was utilized to detect syndrome co‐occurrence patterns. Analyses were stratified by four disease stages, six geographic regions and three periods (2000–2009, 2010–2019, post‐2020). Meta‐analysis and clustering results revealed that blood stasis, qi deficiency and phlegm emerged as conserved core syndrome elements across all stages, dynamically interacting with qi‐yin dual deficiency and phlegm–turbidity as the disease progressed. Geospatial analysis identified a Northern China predominance of qi stagnation‐blood stasis, whereas humid subtropical regions exhibited accelerated phlegm–stasis synergy. Temporal analysis uncovered a paradigm shift toward blood stasis–phlegm synergy, coinciding with modern dietary transitions and sedentary lifestyle trends. By leveraging AI‐driven methodologies, this study reveals the spatiotemporal heterogeneity of CHD syndrome patterns and underscores the necessity for geographically personalized and temporally adaptive TCM therapeutic strategies for CHD management.

## 1 Introduction

Coronary heart disease (CHD) represents a significant public health challenge in China, with urban and rural mortality rates having escalated to 135.08 and 148.19 per 100,000 respectively, alongside a national prevalence of 758/100,000. The direct hospitalization burden reached ¥93.18 billion ($13.6 billion) in 2022, accounting for 43.9% of total cardiovascular hospitalization expenditures [1]. A growing body of evidence indicates that traditional Chinese medicine (TCM) can alleviate symptoms in CHD patients and slow or even halt disease progression through two foundational therapeutic principles: “Syndrome Differentiation” (known as “Bian Zheng Lun Zhi” in Chinese) and “Treatment in Accordance with Three Categories of Etiologic Factors (San Yin Zhi Yi)” [2, 3, 4]. The comprehensive delineation of syndrome patterns across temporal (disease phases), spatial (geographic regions), and pathological progression domains is therefore imperative for developing precision TCM interventions, thereby facilitating early therapeutic strategies to potentially arrest and reverse disease progression [5, 6].

Previous investigations utilizing cohort studies and cross‐sectional designs have attempted to delineate TCM syndrome distribution patterns in CHD; however, persistent discrepancies across studies remain unresolved [7, 8]. A multicenter epidemiological investigation identified blood stasis (77.89%), qi deficiency (67.17%), phlegm–turbidity (43.97%), and yin deficiency (28.97%) as predominant syndrome elements in angina pectoris [9], while a nationwide analysis of 8129 patients by Zhang et al. identified distinct geographic distribution patterns: qi deficiency and blood stasis predominated in Northeast China (77.89%), contrasting with phlegm–turbidity clustering in Southwest/South China and yin deficiency prevalence gradients [10]. Multiple cross‐sectional studies led by Hu et al. demonstrated the progressive accumulation of phlegm‐dampness and blood stasis elements across CHD stages [11]. Current investigations, however, remain impeded by systemic bottlenecks, most notably the prevalence of truncated observation windows (typically ≤12 months) and statistically underpowered cohorts (averaging merely 273 participants). These limitations are frequently compounded by structural methodological deficits—such as regional fragmentation and non‐representative sampling—which collectively obscure the broader epidemiological landscape. Most crucially, the absence of systematic analysis of syndrome progression dynamics impedes the translation of TCM theoretical frameworks into stage‐specific clinical protocols [12].

While individual clinical studies on TCM syndromes inherently suffer from methodological constraints, the systematic integration of cross‐stage evidence can mitigate single‐study biases and enhance evidentiary robustness, ultimately enabling comprehensive syndrome–disease mapping. The advent of digital publishing has precipitated exponential growth in TCM syndrome research. As of 2024, the CNKI database alone indexes over 70,000 articles containing “coronary heart disease syndromes,” presenting both opportunities and analytical challenges [13, 14]. Given that screening the entire research literature on CHD syndromes is error‐prone and extremely time‐intensive, scholars often develop narrow searches focused on one disease stage or limited spatiotemporal range [13, 15, 16]. Current meta‐analyses in CHD syndrome research exhibit fundamental methodological flaws in aggregating syndrome elements and patterns; some reviewed studies inappropriately sum raw frequency counts without statistical normalization or heterogeneity adjustments. Moreover, conventional meta‐analytic approaches are fundamentally limited to quantifying element prevalence rates, failing to capture both the interconnected network relationships among syndrome elements and their dynamic progression patterns across disease stages—thus overlooking the multidimensional complexity inherent in syndrome dynamics. Our previous study demonstrated that the constrained Sequential PAttern Discovery using Equivalence classes (cSPADE) algorithm could effectively identify core syndrome element clusters and progression pathways through frequency pattern mining of clinical trial publications [17].

To address these challenges, we introduced an integrated analytical framework combining artificial intelligence (AI)‐based data mining tools with advanced statistical modeling techniques. By leveraging automated text mining, similarity algorithms, and natural language processing (NLP), we efficiently processed the large‐scale TCM literature, extracting and organizing critical syndrome‐related data. This study further developed a comprehensive analytical paradigm incorporating Bayesian meta‐regression, hierarchical clustering, and cSPADE algorithms to decode the spatiotemporal heterogeneity and stage‐specific syndromic evolution in CHD. This CHD‐focused semantic architecture enabled systematic aggregation of syndrome element knowledge from 651 studies spanning 2000–2023, thereby establishing essential data infrastructure for subsequent spatiotemporal evolution analysis.

## 2 Methods

### 2.1 Search Strategy and Selection Criteria

We searched the following databases from January 1, 2000, to December 31, 2023: Wanfang, CNKI, VIP, PubMed, Embase, MEDLINE, and The Cochrane Library. The search strategy combined title terms for CHD‐related diseases and interventions (e.g., “myocardial ischemia,” “unstable angina,” “acute coronary syndrome,” “percutaneous coronary intervention”), subject filters for TCM‐related terms (e.g., “Chinese medicine,” “herbal medicine,” “acupuncture”), and free‐text terms for syndrome patterns (e.g., “syndrome differentiation,” “syndrome pattern,” “syndrome element”). The review protocol, including the complete search strategies, was pre‐registered in PROSPERO (CRD42025640415). Equivalent strategies were adapted for each database. Studies were considered eligible if they included at least 30 participants who were diagnosed with CHD including myocardial infarction (MI), acute or chronic CHD, and heart failure caused by CHD. All included studies must have reported quantitative TCM syndrome/syndrome element frequencies. Exclusion criteria included: (1) studies that focused exclusively on patients with specific syndromes or included patients with different syndromes in fixed proportions; and (2) studies with overlapping data.

**FIGURE 1:**
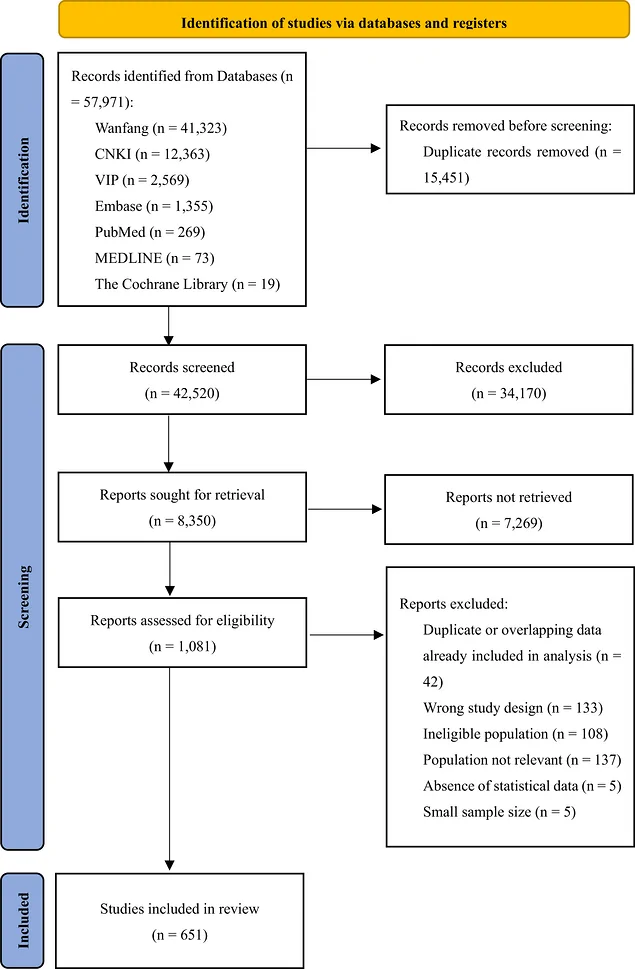
Workflow figure of the study.

An automated pipeline consisted of three critical phases: Selenium WebDriver initially captured 57,971 publications from three databases, which were subsequently reduced to 8350 non‐redundant publications through cosine similarity‐based deduplication (title/abstract terms threshold = 0.85) and metadata cross‐validation. Full‐text mining integrated Tabular PDF processing with YOLOv5 object detection and TableTransformer layout recognition, coupled with optical character recognition, identifying 1081 candidates containing quantitative syndrome distributions. Six reviewers independently conducted independently screening blinded to each other's decisions, via a customized online platform (http://47.95.116.196:40006). Discrepancies underwent senior investigator adjudication, resulting in 651 included studies (Figure 1). The dataset of included study characteristics is available from the corresponding author upon reasonable request.

### 2.2 Data Extraction

For syndrome pattern and element identification, we utilized Word2Vec and Bidirectional Encoder Representations from Transformers (BERT）BERT models to calculate semantic similarity against a standardized dictionary referencing clinical guidelines [18, 19, 20, 21]. To systematically extract structured data, we implemented a semi‐supervised learning framework. A hybrid BERT‐based model integrating W2NER and HGN architectures was developed for baseline information extraction. For syndrome element extraction, a multi‐stage pipeline was employed: table detection via convolutional neural networks achieved 93.4% precision in PDF parsing; rule‐engine guided OCR coupled with BERT‐base‐Chinese embeddings enabled semantic alignment between extracted terms and standardized dictionaries, with 91.2% precision in syndrome element standardization. Cosine similarity thresholds (>0.85) were applied to validate syndrome–element mappings against expert‐curated templates. All extracted data underwent iterative validation by three clinicians with over 10 years’ experience in TCM. Dual independent extraction with automated validation checks ensured data fidelity, with discrepancies resolved through senior investigator adjudication. The extracted syndromes were categorized into two main classes: 23 nature syndrome elements and 15 location syndrome elements. The nature syndrome elements included phlegm, cold, qi stagnation, blood stasis, and others. The location syndrome elements included the heart, lung, liver, spleen, and others.

The extracted cases were classified according to our four‐stage CHD progression framework, defined as follows: (1) Stable stage: stable angina pectoris (CCS Classes I–II), ischemic cardiomyopathy with LVEF ≥40%, or asymptomatic ischemia confirmed by stress testing; (2) Active stage: acute coronary syndrome presentations including unstable angina, NSTEMI, or STEMI, and could include concomitant cerebrovascular events within 30 days post‐ACS or sudden cardiac death; (3) Post‐interventional stage: patients within 180 days after PCI with drug‐eluting stents or CABG; (4) End stage: chronic heart failure caused by CHD meeting NYHA Classes III–IV criteria or LVEF ≤35% on echocardiography. Studies enrolling mixed stages without subgroup reporting were classified as “mixed stage.”

Study locations were stratified into six distinct geographic regions, namely North China, Northeast China, East China, South Central China, Southwest China, and Northwest China, following the National Bureau of Statistics' territorial division standards (GB/T 2260–2022), while temporal trends were analyzed across three time periods (2000–2009, 2010–2019, 2020–2023) to account for healthcare policy cycles. We then compared and analyzed the distribution characteristics of syndrome elements across these spatiotemporal groups. Given the limited number of studies addressing certain syndrome elements, particularly location syndrome elements which appeared in fewer than five publications in most strata, we excluded nature syndrome elements with insufficient literature support (fewer than five publications) and all location syndrome elements from the stratification analyses to ensure robust statistical inference and meaningful interpretation of results.

### 2.3 Quality Assessment

Six independent reviewers assessed the quality of all included studies. The Cochrane Risk of Bias 2 (ROB 2) tool was used for randomized controlled trials (RCTs), the Newcastle–Ottawa Quality Assessment Scale (NOS) for cohort and case‐control studies, and the Joanna Briggs Institute (JBI) critical appraisal tool for cross‐sectional studies and case series, in accordance with established methodological standards [22, 23, 24].

### 2.4 Meta‐Analysis

The pooled prevalence of syndrome elements was estimated using random‐effects meta‐analysis with maximum likelihood estimation, incorporating the logit‐transformed proportions and their standard errors. Stratified analyses were conducted across prespecified dimensions: geographic regions, temporal epochs, and disease stages. Between‐study heterogeneity was explored using a meta‐analytic random‐ effects model with maximum likelihood estimation.

### 2.5 Syndrome Elements Cluster Analysis

Hierarchical clustering was performed to construct dendrograms, with similarity matrices between clusters calculated using the agglomerative method to generate corresponding heatmaps. These heatmaps visually represented syndrome element co‐occurrence frequency and similarity through color gradients, where darker colors indicate higher similarity. Core syndrome element clusters were identified based on abrupt cut‐offs in color gradients and the proximity of critical branches in the dendrogram. When a significant discontinuity in color intensity aligned with a critical dendrogram branch point, indicating a marked reduction in inter‐cluster similarity, the dendrogram was truncated at this position. The retained clusters corresponding to higher similarity values (darker regions in the heatmap) were defined as core syndrome element clusters, representing combinations of syndrome elements with strong inter‐element similarity and frequent co‐occurrence. All analyses were executed in R 4.2.0 (metafor package), and Python 3.10 for cSPADE implementation [25].

### 2.6 Syndrome Elements Sequence Analysis

Based on our prior validation of symptom temporal progression modeling, we adapted the cSPADE algorithm to decode CHD syndrome element trajectories through three key theoretical innovations [17]. The underlying assumption was that syndrome elements appearing earlier in disease progression would show higher prevalence across subsequent stages. Implementing depth‐first search with stage‐adaptive minimum support thresholds (0.1), the algorithm identified significant syndrome transitions (A→B) by computing confidence as follows:

$$\mathrm{Confidence}\;\left(A\to B\right)=P\;\left(B|A\right)=\frac{\mathrm{Support}\left(A\to B\right)}{\mathrm{Support}\left(A\right)}$$

$$\mathrm{Lift}\left(A\to B\right)=\frac{P\left(B|A\right)}{P\left(B\right)}=\frac{\mathrm{Confidence}\left(A\to B\right)}{\mathrm{Support}\left(B\right)}$$

Rules were retained if the lift value was greater than 1 [11]. Hasse diagram visualization incorporated force‐directed layouts optimized for clinical interpretability. Node radii were logarithmically scaled with element prevalence, and edge thickness encoded transition strength through confidence‐lift products. Rules with lift values less than one were excluded from the visualization.

## 3 Results

### 3.1 Characteristics of CHD Syndrome Studies in China

In total, 57,971 titles were identified through database searches, of which 651 were included in this review (Figure 1). A total of 234,656 participants were included in this analysis, with the number of subjects per study from 35 to 22,853. The average age of participants across studies ranged from 42 to 82 years. This analysis included studies of patients in the Stable stage (*n* = 54), Active stage (*n* = 156), Post‐interventional stage (*n* = 42), End stage (*n* = 25) and Mixed stage (*n* = 377) patients. Three studies enrolled patients spanning two stages and thus contributed to both groups, resulting in the total count of categorized studies exceeding the actual number of unique publications by two. The included studies originated from six regions of China, with the distribution of papers by region shown in Table 1. The temporal distribution showed most studies published between 2010 and 2019, whereas studies from 2000–2009 contributed most cases (137,390, 58.55%).

**TABLE 1:** Distribution of CHD stages included in the literature.

Group	Value	Number of articles	Sample size (%)
CAD stage	Stable stage	54	21,895 (9.33%)
	Active stage	156	29,998 (12.78%)
	Post‐interventional stage	42	11,941 (5.09%)
	End stage	25	4906 (2.09%)
	Mixed	376	165,916 (70.71%)
Region	East China	144	32,604 (13.89%)
	North China	136	78,783 (33.57%)
	Northeast China	122	35,615 (15.18%)
	Northwest China	51	26,611 (11.34%)
	South Central China	146	29,228 (12.46%)
	Southwest China	26	3985 (1.70%)
	Missing	26	27,830 (11.86%)
Period	2000–2009	194	75,233 (32.06%)
	2010–2019	368	137,390 (58.55%)
	2020–2023	89	22,033 (9.39%)
Total		651	234,656

The RoB 2 assessment indicated that while most RCTs exhibited a moderate risk of bias, concerns were identified in areas such as protocol deviations, outcome measurement, and selective reporting for some studies. The NOS assessment showed that the majority of cohort and case‐control studies were of good quality, with some case‐control studies demonstrating only fair quality regarding selection of controls. The JBI assessment revealed that most cross‐sectional studies were of good quality, while approximately half of the included case series demonstrated relatively high risk of bias in reporting outcomes of follow‐up. The detailed risk of bias assessment results, including the summary figures, are not presented here due to legibility constraints but are available from the corresponding author upon reasonable request.

**FIGURE 2:**
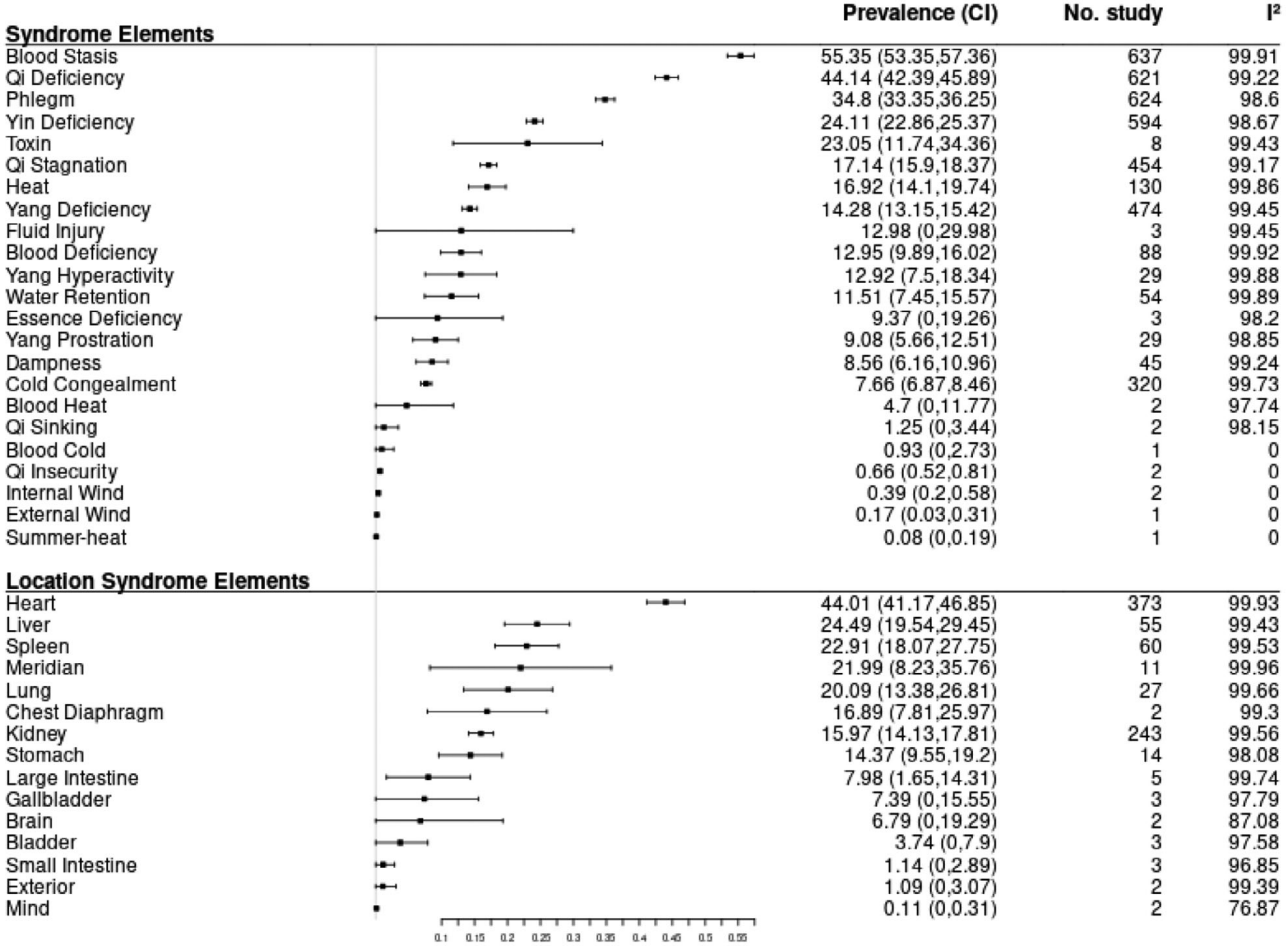
Prevalence of syndrome elements (ranked) in the total population. In total 38 syndrome elements were reported.

The meta‐analysis of 234,656 patients yielded pooled prevalence estimates for various syndrome elements. Blood stasis was the most common syndrome element, reported in 55.35% of patients (95% confidence interval [CI] 53.35%–57.36%; 637 studies), followed by qi deficiency (44.14%; 95% CI 42.39%–45.89%; 621 studies), phlegm (34.8%; 95% CI 33.35%–36.25%; 624 studies), yin deficiency (24.11%; 95% CI 22.86%–25.37%; 594 studies), and toxin (23.05%; 95% CI 11.74%–34.36%; 8 studies). Other syndrome elements ranged in prevalence from qi stagnation at 17.14% to summer heat at 0.08%. A comprehensive summary of all syndrome elements and their respective prevalence rates from the meta‐analysis is presented in Figure 2.

### 3.2 CHD Stage‐Stratified Differences in Syndrome Element Clustering and Transition Sequences

**FIGURE 3:**
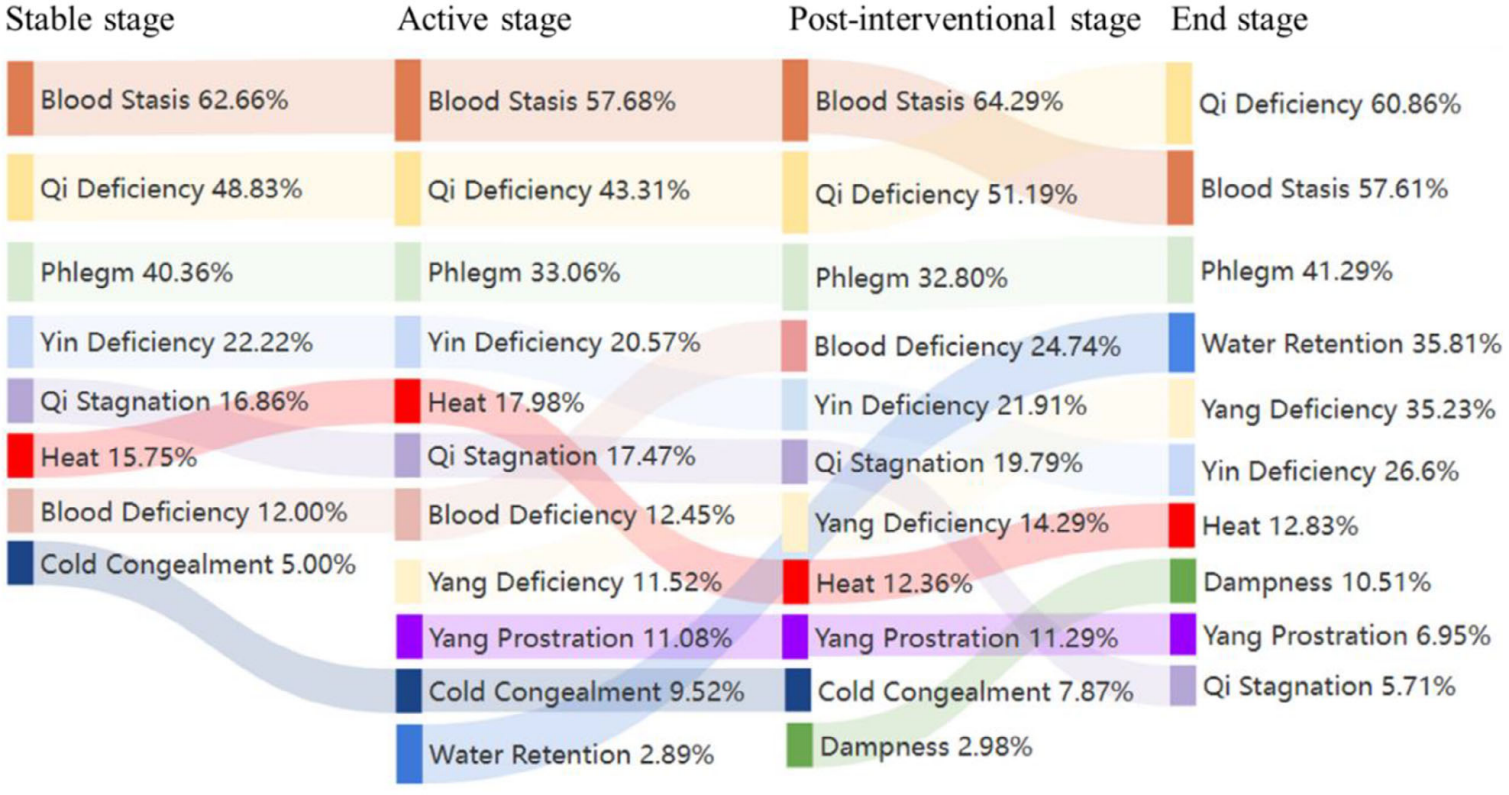
Temporal shifts in nature syndrome element prevalence rankings across disease progression stages.

**FIGURE 4:**
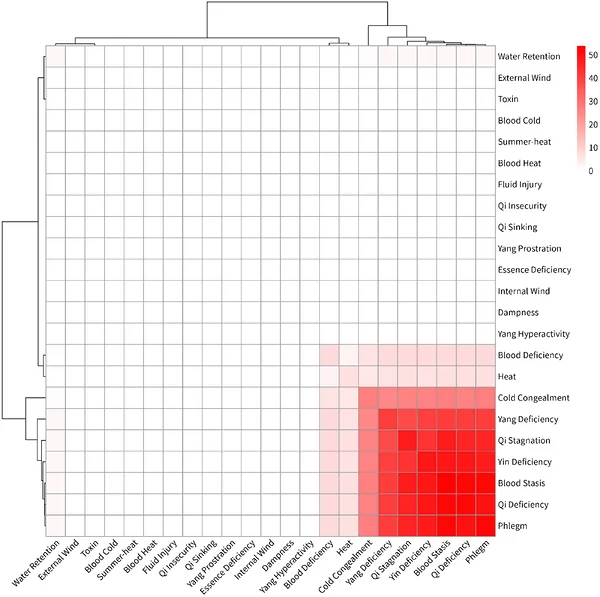
Visualization of Stable stage syndrome element clusters derived from agglomerative hierarchical clustering. Heatmaps encode co‐occurrence frequencies through color gradients (darker hues = higher similarity), with dendrograms illustrating cluster hierarchy.

Meta‐analysis and hierarchical cluster analysis of syndrome element co‐occurrence matrices revealed a set of conserved core elements across the stable, active, and post‐interventional stage of CHD (Figure 3). These core elements comprised cold congealment, yang deficiency, qi stagnation, yin deficiency, blood stasis, qi deficiency, and phlegm, while dampness emerged as a unique feature exclusively in end‐stage cohorts. Stable stage patients exhibited tightly interconnected syndrome networks excluding cold congealment (4.96%; 95% CI 2.19%–7.72%; 27 studies), which displayed weaker associations with other elements (Figure 4). Active stage clusters bifurcated into two distinct subgroups: one anchored by cold congealment (9.52%; 95% CI 7.93%–11.11%; 84 studies), yang deficiency (11.52%; 95% CI 10.14%–12.91%; 111 studies), and qi stagnation (17.47%; 95% CI 14.85%–20.08%; 106 studies); and another dominated by yin deficiency (20.57%; 95% CI 18.32%–22.88%; 136 studies), blood stasis (57.68%; 95% CI 53.46%–61.89%; 151 studies), qi deficiency (44.00%; 95% CI 40.42%–47.58%; 142 studies), and phlegm (33.05%; 95% CI 30.33%–35.78%; 150 studies), reflecting divergent pathological trajectories (Figure S1).

Post‐interventional stages further fragmented these associations, isolating cold congealment (7.87%; 95% CI 4.42%–11.33%; 22 studies) while segregating qi deficiency (51.19%; 95% CI 44.27%–58.12%; 41 studies), blood stasis (64.29%; 95% CI 57.51%–71.08%; 41 studies), phlegm (32.80%; 95% CI 26.31%–39.30%; 41 studies), and yin deficiency (21.91%; 95% CI 15.30%–28.53%; 35 studies), yang deficiency (14.29%; 95% CI 8.07%–20.51%; 32 studies), qi stagnation (19.79%; 95% CI 13.21%–26.36%; 34 studies) into separate clusters (Figure S1). End‐stage patients manifested a paradigm shift, with phlegm (41.29%; 95% CI 31.26%–51.31%; 16 studies) and dampness (10.51%; 95% CI 5.72%–15.29%; 13 studies) forming a dominant cluster exhibiting strong co‐occurrence, whereas qi deficiency (60.86%; 95% CI 50.58%–71.15%; 22 studies), yin deficiency (26.60%; 95% CI 22.08%–31.12%; 23 studies), and yang deficiency (35.23%; 95% CI 27.51%–42.96%; 22 studies) coalesced into a secondary group (Figure S1).

**FIGURE 5:**
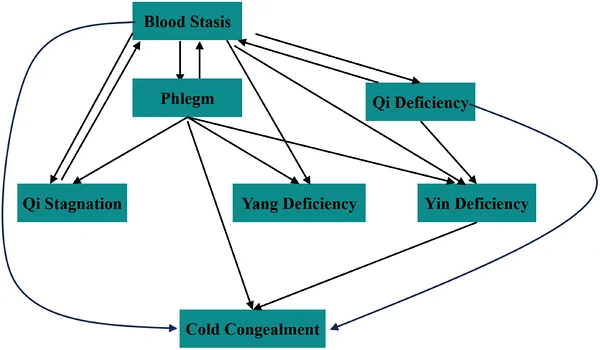
Results from the cSPADE algorithm on Stable stage of CHD with 0.1 and above support and confidence.

The cSPADE‐derived transition sequences revealed stage‐dependent progression pathways aligned with TCM pathogenesis principles. The cSPADE analysis identified blood stasis (support = 0.56, confidence = 0.63) and qi stagnation as dominant syndrome elements in stable‐stage CHD patients, exhibiting bidirectional transitions that aligned with the traditional pathomechanism of “stasis causing stagnation, stagnation exacerbating stasis” (Figure 5). A substantial subset (support = 0.40) progressed from blood stasis–qi deficiency complexes to either yin deficiency (confidence = 0.62) or qi stagnation recurrence (confidence = 0.62). Blood stasis further evolved into yang deficiency (support = 0.37, confidence = 0.41) or mediated phlegm–stasis intermingling pathways (support = 0.35, confidence = 0.62) that precipitated secondary qi stagnation. Active‐stage patients predominantly transferred from blood stasis to yin deficiency (support = 0.43, confidence = 0.54), aligning with the “prolonged stasis transforming into heat injuring yin” pathogenesis (Figure S2). Concurrently, qi‐yin deficiency complexes emerged as critical nodal points, either de novo (support = 0.40, confidence = 0.60) or evolving from blood stasis precursors (support = 0.34, confidence = 0.68). While bidirectional blood stasis–qi stagnation transitions persisted (support = 0.29, confidence = 0.37), their prevalence was reduced compared to stable stages.

Post‐interventional stage trajectories revealed qi‐yin dual deficiency as the predominant syndrome complex (support = 0.30, confidence = 0.43). Residual blood stasis pathways demonstrated attenuated progression to yang deficiency (support = 0.23, confidence = 0.28), while qi deficiency mediated cold congealment transitions (support = 0.18, confidence = 0.23). Concurrently, qi deficiency–phlegm turbidity complexes progressed to cold congealment (support = 0.20, confidence = 0.29 and support = 0.18, confidence = 0.28, respectively) (Figure S2). End‐stage trajectories demonstrated concentrated qi‐yin dual deficiency patterns (support = 0.52, confidence = 0.72), epitomizing the Huangdi Neijing's principle of “prolonged illness manifests deficiency.” Qi deficiency systematically progressed to phlegm–turbidity (support = 0.52, confidence = 0.72) and subsequently to yin deficiency (support = 0.32, confidence = 0.89). Blood stasis (support = 0.44, confidence = 0.92) and phlegm–turbidity (support = 0.36, confidence = 0.82) trajectories converged on yin deficiency (Figure S2).

### 3.3 Geographic Region‐Specific Variations in Syndrome Element Clustering and Transition Sequences

**FIGURE 6:**
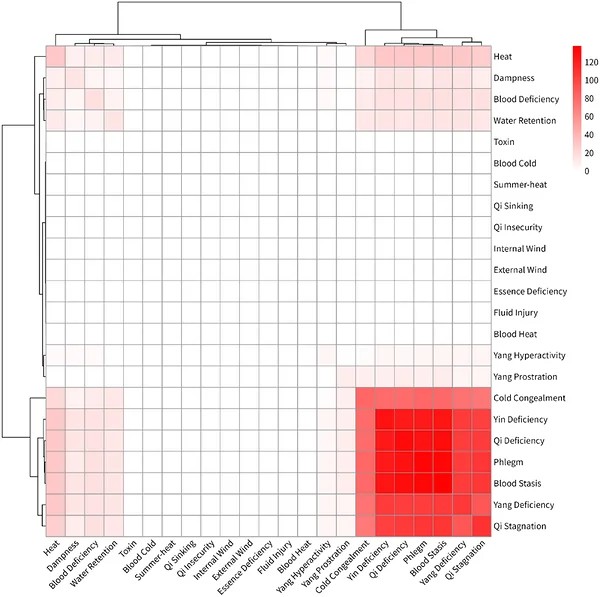
Visualization of North China syndrome element clusters derived from agglomerative hierarchical clustering. Heatmaps encode co‐occurrence frequencies through color gradients (darker hues = higher similarity), with dendrograms illustrating cluster hierarchy.

Cluster analysis under geographic stratification revealed conserved core syndrome elements consistent with those identified in temporal and stage‐stratified clusters. In North China, syndrome networks partitioned into two distinct clusters: the first centered on yang deficiency (16.35%; 95% CI 13.99%–18.72%; 107 studies) and qi stagnation (15.20%; 95% CI 12.95%–17.44%; 110 studies), and the second characterized by coordinated manifestation of qi deficiency (47.87%; 95% CI 44.23%–51.51%; 132 studies), yin deficiency (24.07%; 95% CI 21.49%–26.66%; 127 studies), phlegm (38.29%; 95% CI 35.25%–41.33%; 134 studies), and blood stasis (63.11%; 95% CI 58.92%–67.31%; 138 studies). Cold congealment exhibited isolated clustering (6.69%; 95% CI 5.18%–8.19%; 82 studies) (Figure 6). Northeast China demonstrated distinct aggregation patterns, eliminating cold congealment as a core element while retaining other syndrome clusters observed in North China (Figure S3).

East China exhibited progressive network simplification, segregating yang deficiency (14.78%; 95% CI 12.54%–17.02%; 99 studies), qi stagnation (18.41%; 95% CI 15.38%–21.45%; 79 studies), and cold congealment (7.25%; 95% CI 5.87%–8.64%; 68 studies) into separate topological domains. Meanwhile, yin deficiency (26.48%; 95% CI 23.85%–29.12%; 133 studies), qi deficiency (41.95%; 95% CI 38.29%–45.62%; 135 studies), phlegm (29.14%; 95% CI 26.52%–31.76%; 134 studies), and blood stasis (48.53%; 95% CI 44.44%–52.62%; 134 studies) maintained characteristic clustering patterns of qi‐yin deficiency and phlegm–stasis binding (Figure S3). South Central China exhibited a similar clustering paradigm to that observed in East China (Figure S3).

In Southwest China, yang deficiency (11.53%; 95% CI 7.61%–15.45%; 16 studies) and qi stagnation (20.62%; 95% CI 15.11%–26.14%; 14 studies) formed a distinct cluster alongside the conserved qi and yin deficiency, and phlegm and stasis binding group (Figure S3). In contrast, Northwest China integrated cold congealment (12.29%; 95% CI 8.85%–15.72%; 26 studies) into the yang deficiency–qi stagnation cluster, highlighting regional climatic modulation of syndrome aggregation (Figure S3).

**FIGURE 7:**
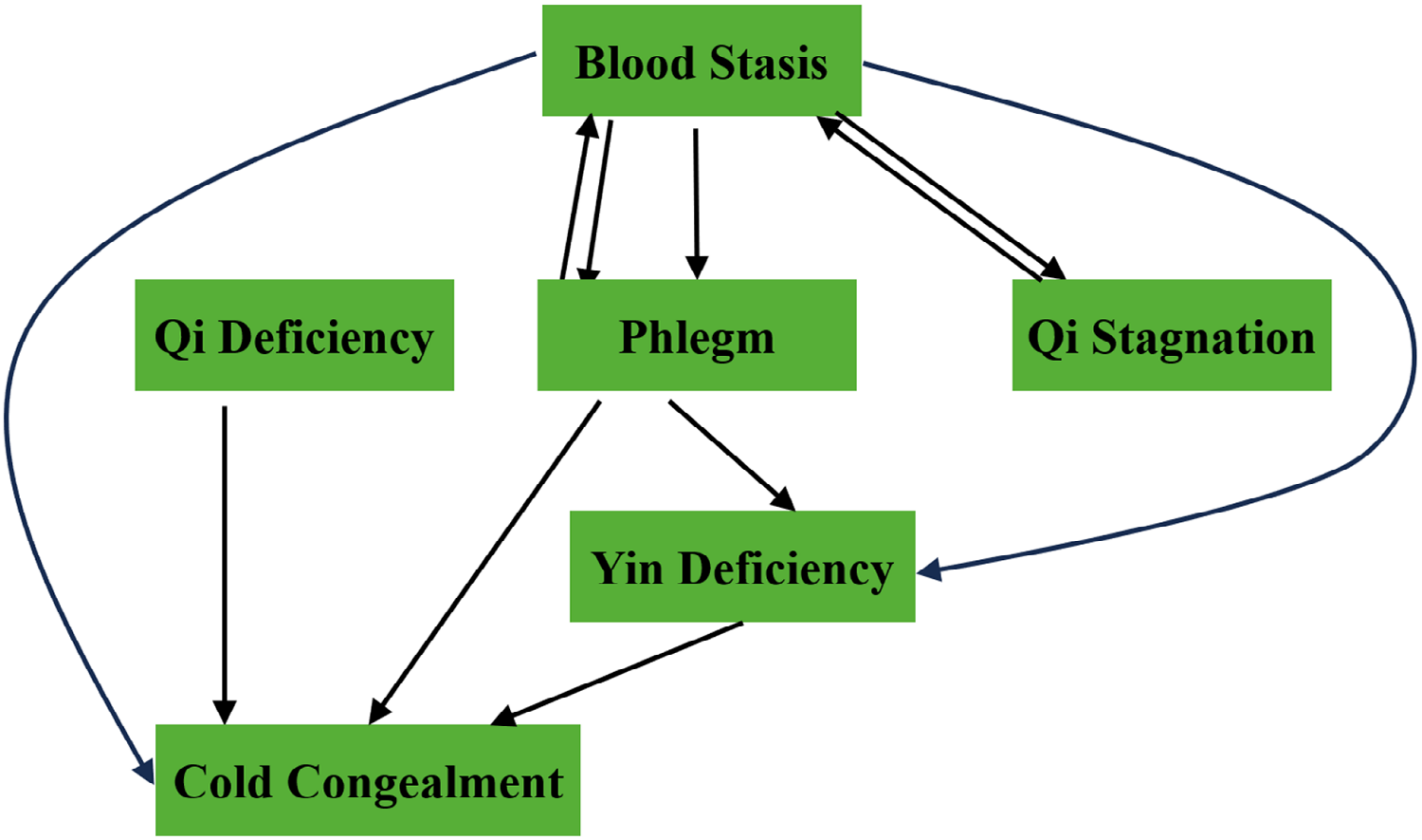
Results from the cSPADE algorithm on North China syndrome element clusters derived from agglomerative hierarchical clustering. Heatmaps encode co‐occurrence frequencies through color gradients (darker hues = higher similarity), with dendrograms illustrating cluster hierarchy.

The cSPADE trajectories in North China identified blood stasis as the primary initiation node (support = 0.40, confidence = 0.69 for blood stasis → qi‐yin dual deficiency, Figure 7). Key progression pathways bifurcated into qi stagnation–blood stasis complexes (support = 0.39, confidence = 0.46) and blood stasis–cold congealment interactions (support = 0.28, confidence = 0.33). Phlegm obstruction pathways (support = 0.26, confidence = 0.47) precipitated secondary qi stagnation. The cSPADE trajectory analysis in Northeast China highlighted phlegm–stasis intermingling as the predominant syndrome complex (support = 0.61, confidence = 0.75). Blood stasis systematically transitioned to qi‐yin dual deficiency (support = 0.33, confidence = 0.59). Distinctively, yang deficiency emerged as a critical convergence node, with trajectories from blood stasis (support = 0.34, confidence = 0.48), qi deficiency (support = 0.32, confidence = 0.52), and phlegm–turbidity (support = 0.31, confidence = 0.45) all culminating in yang deficiency.

In East China, cSPADE trajectory analysis identified qi‐yin deficiency as the predominant syndrome (support = 0.38, confidence = 0.56, Figure S4), while progressive qi‐yang deficiency pathways (support = 0.25, confidence = 0.37) validated the coastal climate adaptation pattern. Blood stasis cases systematically progressed to qi‐yin dual deficiency (support = 0.24, confidence = 0.60), cold congealment (support = 0.23, confidence = 0.31), and qi stagnation (support = 0.22, confidence = 0.30), reflecting the pathomechanisms characteristic of humid subtropical regions. In South Central China, cSPADE trajectory analysis demonstrated that progression from qi deficiency to qi‐yin dual deficiency constituted the dominant pathway (support = 0.37, confidence = 0.60, Figure S4). Blood stasis systematically evolved into qi‐yin dual deficiency (support = 0.24, confidence = 0.66) or yin deficiency (support = 0.36, confidence = 0.51), quantifying the mechanism of “chronic stasis transforming heat, scorching genuine yin” prevalent in subtropical climates. Bidirectional blood stasis–qi stagnation interactions were also observed (support = 0.24, confidence = 0.34), while phlegm–turbidity frequently transferred to qi stagnation (support = 0.24, confidence = 0.38).

The cSPADE trajectories in Southwest China revealed unique progression dynamics, where phlegm–turbidity systematically transitioned to yang deficiency (support = 0.31, confidence = 0.42, Figure S4) or dual yin‐yang deficiency (support = 0.15, confidence = 0.50), validating high‐altitude pathophysiology. Blood stasis cases predominantly evolved into qi stagnation (support = 0.31, confidence = 0.44) or yang deficiency (support = 0.27, confidence = 0.39), with a subset progressing through phlegm–turbidity intermediaries to yang deficiency (support = 0.19, confidence = 0.42). In Northwest China cSPADE trajectory analysis revealed distinct arid‐climate adaptation patterns, where blood stasis predominantly progressed to qi stagnation (support = 0.27, confidence = 0.37, Figure S4). Yin deficiency cases systematically transitioned to qi stagnation (support = 0.20, confidence = 0.37). Qi deficiency pathways mediated through phlegm–turbidity intermediaries culminated in qi‐yin dual deficiency (support = 0.16, confidence = 0.67).

### 3.4 Epoch‐Specific Differences in Syndrome Element Clustering and Transition Sequences Across Study Periods

**FIGURE 8:**
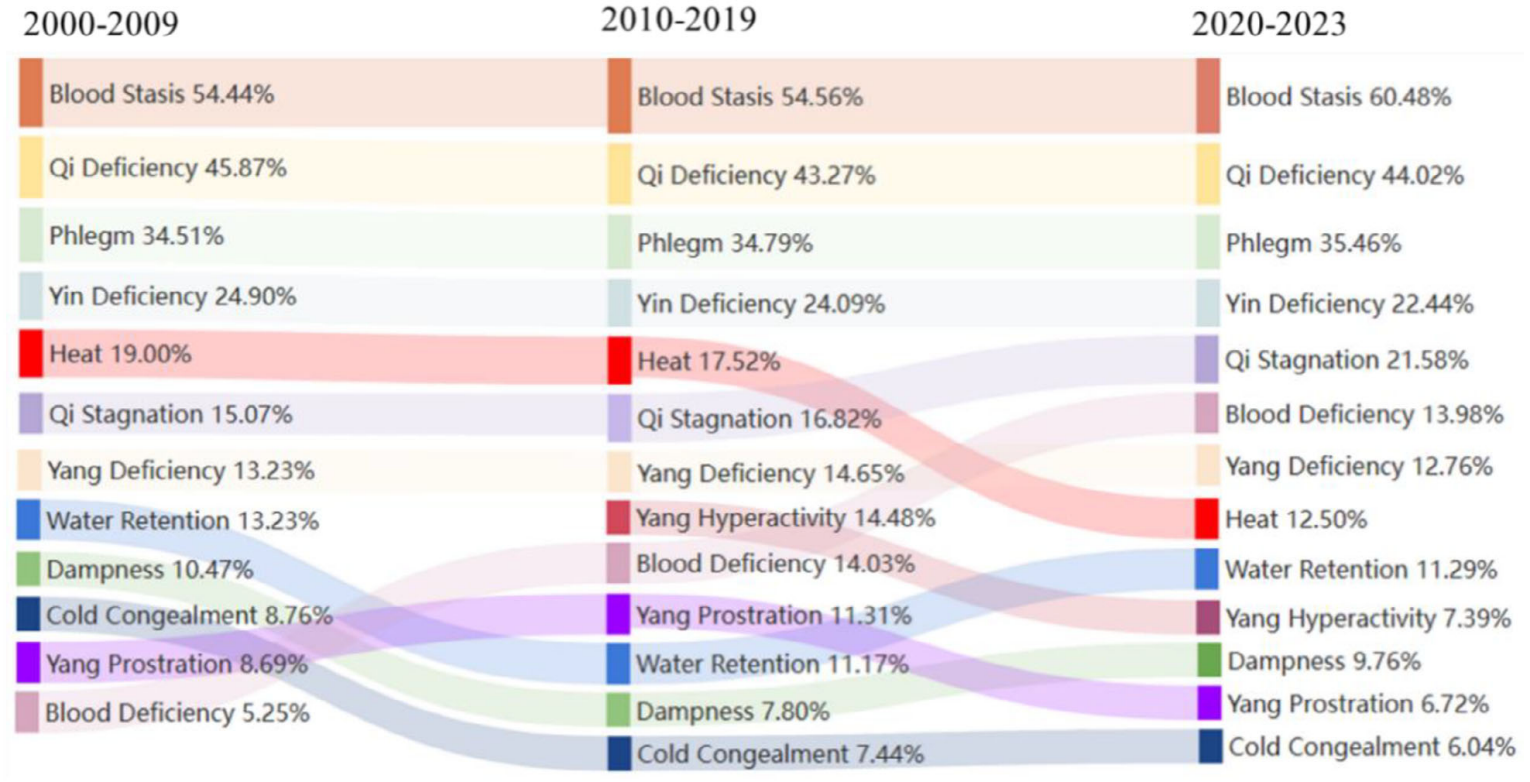
Temporal shifts in nature syndrome element prevalence rankings across three epochs (2000–2023).

**FIGURE 9:**
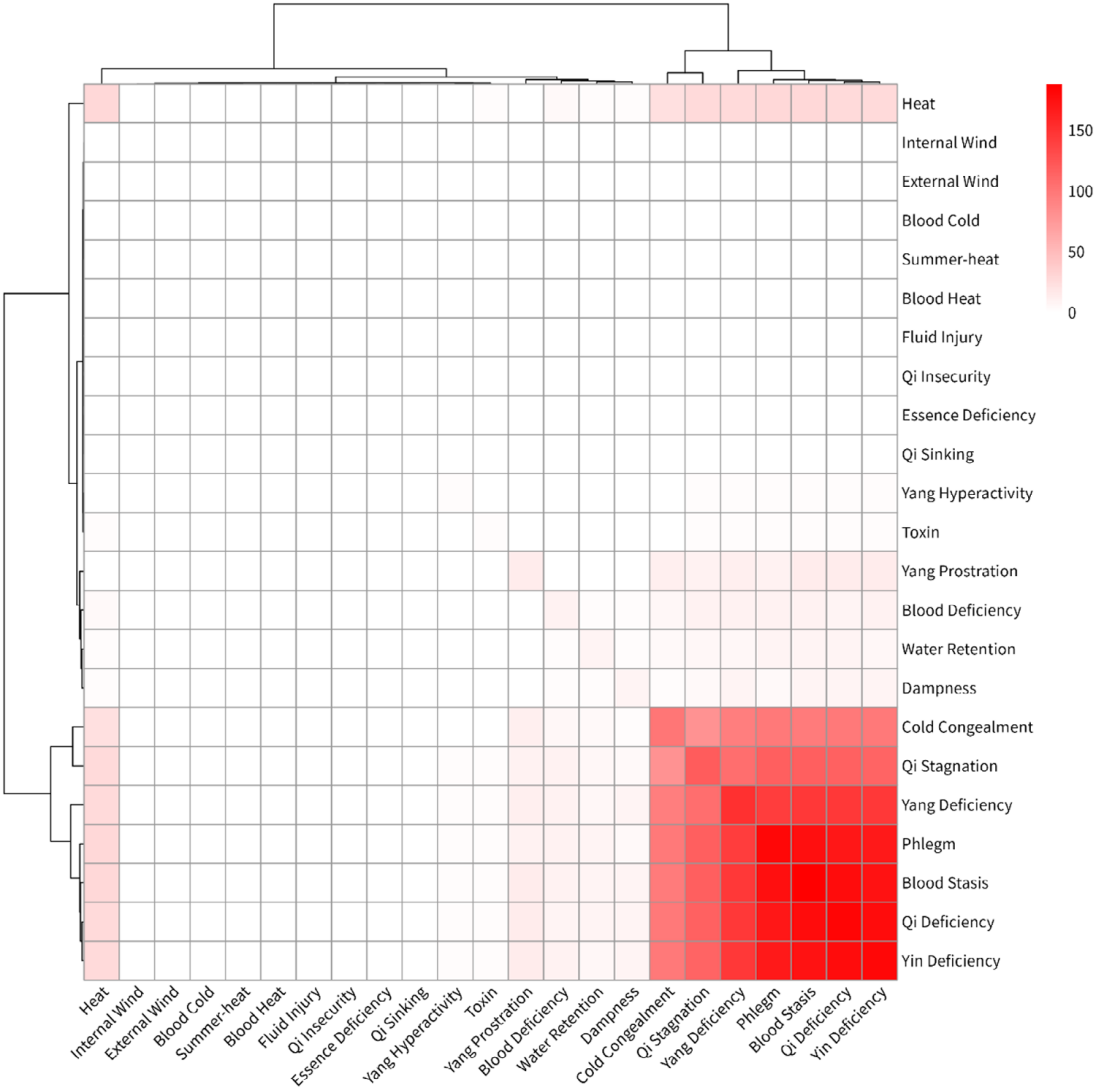
Visualization of 2000–2009 syndrome element clusters derived from agglomerative hierarchical clustering. Heatmaps encode co‐occurrence frequencies through color gradients (darker hues = higher similarity), with dendrograms illustrating cluster hierarchy.

Meta‐analysis and hierarchical cluster analysis under period stratification revealed conserved core syndrome elements consistent with those identified in CHD stage‐stratified clusters, with the exception of dampness (Figure 8). During 2000–2009, syndrome networks partitioned into dual configurations: the first centered around cold congealment (8.76%; 95% CI 7.15%–10.37%; 101 studies, Figure 9) and qi stagnation (15.07%; 95% CI 12.68%–17.45%; 120 studies), and the second characterized by coordinated manifestation of yang deficiency (14.12%; 95% CI 12.58%–15.67%; 151 studies), phlegm (34.51%; 95% CI 31.76%–37.26%; 182 studies), blood stasis (54.44%; 95% CI 50.42%–58.47%; 188 studies), qi deficiency (45.87%; 95% CI 42.49%–49.25%; 184 studies), and yin deficiency (24.90%; 95% CI 22.63%–27.17%; 181 studies). The 2010–2019 period demonstrated progressive network disaggregation, with cold congealment (7.44%; 95% CI 6.40%–8.48%; 182 studies, Figure S5) evolving as an independent node. Concurrently, yang deficiency (14.65%; 95% CI 12.96%–16.35%; 265 studies), qi stagnation (16.82%; 95% CI 15.19%–18.44%; 260 studies), yin deficiency (24.09%; 95% CI 22.41%–25.77%; 334 studies), qi deficiency (43.27%; 95% CI 41.00%–45.55%; 349 studies), phlegm (34.79%; 95% CI 32.88%–36.70%; 355 studies), and blood stasis (54.56%; 95% CI 52.01%–57.11%; 360 studies) established distinct topological domains. Post‐2020 patients exhibited a distinctive syndrome pattern characterized by yin deficiency (22.44%; 95% CI 19.07%–25.82%; 79 studies, Figure S5) and qi stagnation (21.58%; 95% CI 18.68%–24.48%; 74 studies) forming one cluster, contrasted by an ancillary cluster comprising phlegm (35.46%; 95% CI 31.64%–39.29%; 87 studies), qi deficiency (44.02%; 95% CI 39.37%–48.67%; 88 studies), and blood stasis (60.48%; 95% CI 55.22%–65.74%; 89 studies). Yang deficiency (12.76%; 95% CI 9.73%–15.79%; 58 studies) persisted as an isolated topological entity, underscoring epoch‐dependent syndromic realignment.

**FIGURE 10:**
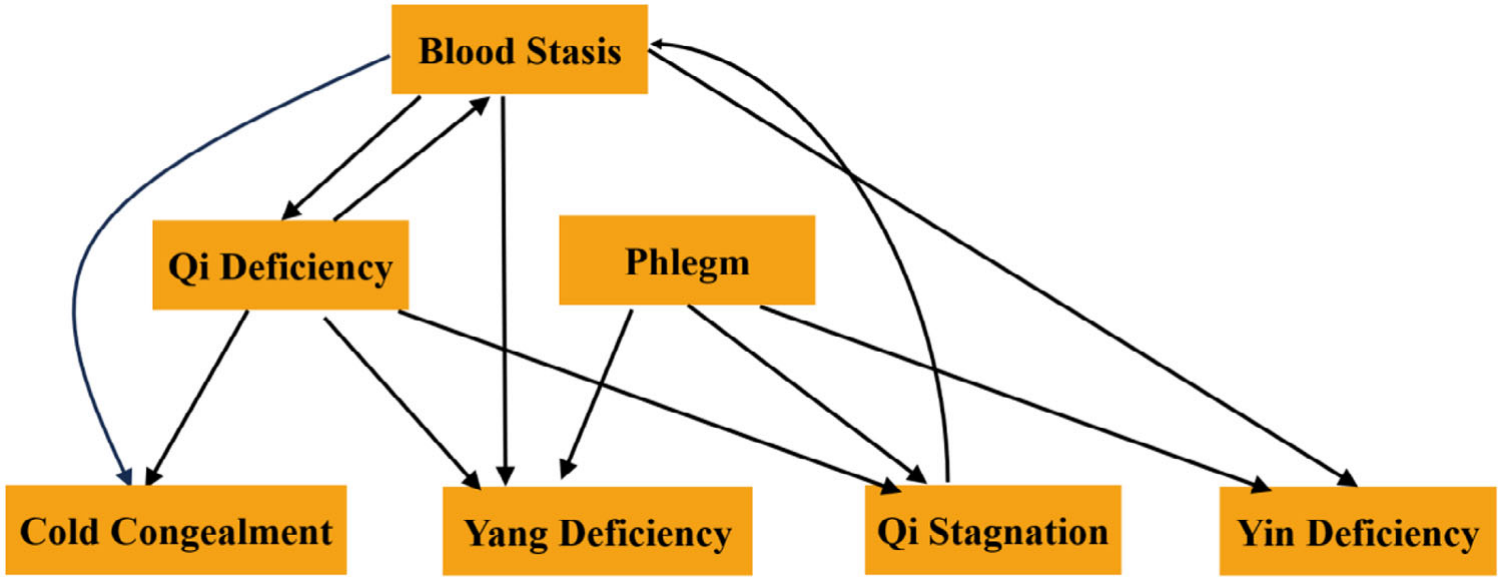
Results from the cSPADE algorithm on CHD during 2000–2009 with 0.1 and above support and confidence.

The cSPADE trajectories during the 2000–2009 epoch revealed qi‐yin dual deficiency as the predominant syndrome complex (support = 0.43, confidence = 0.54), while blood stasis systematically progressed to qi‐yin deficiency (support = 0.32, confidence = 0.70, Figure 10). Concurrently, qi deficiency (support = 0.28, confidence = 0.41) and phlegm–turbidity (support = 0.27, confidence = 0.42) pathways converged on yang deficiency. Transition patterns identified by the cSPADE algorithm during 2010–2019 highlighted qi‐yin dual deficiency as the central syndrome complex (support = 0.38, confidence = 0.56, Figure S6). Qi deficiency pathways emerged as predominant drivers of yang deficiency evolution (support = 0.24, confidence = 0.36), while blood stasis (support = 0.30, confidence = 0.38) and phlegm–turbidity (support = 0.27, confidence = 0.39) trajectories predominantly precipitated qi stagnation. A clinically significant subset demonstrated blood stasis → qi‐yin dual deficiency transitions (support = 0.26, confidence = 0.58). The 2020–2023 period analysis revealed a paradigm shift in progression pathways, with blood stasis emerging as the predominant initiation node (support = 0.58, confidence = 0.73 for blood stasis → phlegm–stasis complexes, Figure S6). Key trajectories bifurcated into qi stagnation–blood stasis interactions (support = 0.51, confidence = 0.63) and yin deficiency–blood stasis complexes (support = 0.46, confidence = 0.58). Notably, 35% of blood stasis cases progressed to qi‐yin dual deficiency (support = 0.35, confidence = 0.61).

## 4 Discussion

Traditional investigations into CHD syndromes often face significant operational challenges, as capturing long‐horizon, multicenter spatiotemporal dynamics is hindered by the high costs of prospective cohorts and the inherent subjectivity of syndrome differentiation. To overcome these barriers, this study applied an AI‐assisted literature mining and data governance approach, converting unstructured evidence into a computable knowledge base. This methodology allowed us to synthesize data from 234,656 patients across a 24‐year span (2000–2023), creating a system‐level characterization of syndromic heterogeneity. Through a novel integration of disease stage stratification, temporal evolution, and geographic specificity, we identified robust distribution patterns: blood stasis, qi deficiency, and phlegm emerged as core anchors, while dynamic modeling revealed stage‐specific trajectories and period‐dependent realignments. By bridging the gap between fragmented clinical data and systemic modeling, this study demonstrates that AI‐driven retrospective mining serves as a powerful, updatable alternative to continuous de novo cohort construction for mapping TCM syndrome evolution.

The predominance of blood stasis (55.27%; 95% CI 53.25%–57.29%) across 234,656 CHD patients in this meta‐analysis substantiates its role as the central pathomechanism in Chinese populations. This finding aligns with the Huangdi Neijing's foundational axiom that “stagnation induces heart pain” and “vessel obstruction leading to blood stasis” [26]. This syndromic centrality persists across all disease stages, geographic regions and temporal epochs, with qi deficiency (43.94%; 95% CI 42.18%–45.71%) and phlegm–turbidity (34.75%; 95% CI 33.30–36.20%) forming consistent co‐pathogenic triads. Contemporary literature further corroborates the primacy of blood stasis in CHD pathogenesis. Niu et al.’s systematic review (1981–2018) identified blood stasis as the predominant syndrome element (30.15%), followed sequentially by qi deficiency (25.92%) and phlegm–turbidity (17.00%) [27]. Notably, Zhong et al.’s recent investigation of 1371 CHD patients demonstrated the consistent predominance of blood stasis, phlegm‐dampness, and qi deficiency across CHD disease stages, with blood stasis–phlegm combinations constituting fundamental syndrome patterns [28]. Emerging evidence delineates a coherent pathophysiological sequence underlying blood stasis syndrome in CHD. Proteomic investigations have identified characteristic molecular signatures differentiating CHD patients with blood stasis from controls, including elevated fibrinogen chains (β, γ), haptoglobin β‐chain, and α1‐antitrypsin concurrent with reduced apolipoproteins (A1, A4) and transthyretin levels [29, 30, 31]. Metabolomic profiling further delineates distinctive perturbations in blood stasis syndrome, manifesting as dysregulated amino acid, lipid, glucose, and purine metabolism, with concomitant elevation of oxygen‐free radicals and marked vasoconstriction predisposing these patients to hepatorenal complications [32]. These dysregulated proteins and metabolites converge on two interconnected pathological axes: inflammatory cascade activation and coagulation system potentiation [33]. Mechanistic studies implicate membrane‐bound subcellular particles in circulation as initiators, releasing signaling molecules that simultaneously trigger NF‐κB‐mediated cytokine production (hs‐CRP, TNF‐α, IL‐6) and thrombin generation through tissue factor exposure [31, 34]. These multidimensional findings collectively reinforce blood stasis as the central pathogenic element in CHD development while highlighting the complexity of interacting syndrome components across disease progression.

Our stage‐stratified analysis elucidates distinct pathophysiological trajectories in CHD progression through the lens of TCM syndrome evolution. The conserved core syndrome elements, including cold congealment, yang deficiency, qi stagnation, yin deficiency, blood stasis, qi deficiency, and phlegm, across stages validate the foundational role of qi‐blood dysfunction in CHD pathogenesis. Crucially, the emergence of dampness in end‐stage cohorts signals the transition from functional disparity to structural “fluid metabolism collapse.” Stable‐stage networks dominated by qi stagnation–blood stasis complexes (cSPADE support = 0.56) manifest the classical “stasis–stagnation mutual exacerbation” principle, where impaired qi movement precipitates blood stasis and vice versa. Biomedically, this corresponds to the early phase of endothelial dysfunction and hemorheological disorders. This mechanistic duality finds biochemical corroboration in studies linking elevated red blood cell distribution width with qi stagnation and blood stasis, mirroring TCM's pathological progression from functional qi disorder to tangible stasis [35]. The prognostic significance of these syndrome factors is further evidenced by their association with increased risk of 1‐year cardiovascular event recurrence in a prospective cohort study among stable‐stage CHD patients [36].

Active‐stage progression from blood stasis to yin deficiency (support = 0.43, confidence = 0.54) empirically verifies the traditional pathomechanism of “prolonged disease entering the collaterals” (Jiu Bing Ru Luo) and “prolonged stasis transforming into heat injuring yin” (Yu Re Shang Yin). This trajectory suggests a shift from vascular occlusion to microcirculatory ischemic damage. Meanwhile, post‐interventional qi‐yin dual deficiency predominance (support = 0.30, confidence = 0.43) reflects iatrogenic qi consumption during revascularization procedures. Contemporary cohort studies and meta‐analyses substantiate these transitions. Acute coronary syndrome patients predominantly exhibited blood stasis, heat–toxin, and phlegm–turbidity complexes with concomitant qi‐yin deficiency [37], while post‐PCI populations demonstrated a 38.89% prevalence of qi deficiency–blood stasis patterns [38]. Mechanistic studies further corroborated these clinical observations: qi deficiency patterns demonstrated decreased complement components (C3, C7) and prothrombin levels, indicating compromised immune competence and coagulation regulation [39], while yin deficiency was associated with altered lipid metabolism markers including apolipoprotein dysregulation [40].

The end‐stage pathogenesis crystallized into a triad of blood stasis, qi deficiency, and dampness accumulation, reflecting the decompensation of chronic cardiometabolic function. Wang et al.’s analysis of 260 acute heart failure (AHF) patients quantifies this progression, revealing blood stasis (66.15%) and qi deficiency (57.31%) as cardinal elements, frequently co‐occurring with dampness (32.69%) as demonstrated through association rule mining [41]. Notably, qi deficiency patients exhibited prolonged prothrombin time (PT: 11.80 vs. 11.40 s, *p* = 0.026) with reduced PT% (86.55 vs. 91.00, *p* = 0.011), biochemical evidence supporting TCM's concept of the “qi failing to command blood” mechanism and indicating heightened bleeding risks requiring qi‐tonifying interventions [42]. Furthermore, patients with dampness syndrome exhibited significantly elevated myocardial stress biomarkers compared to non‐dampness patients, including brain natriuretic peptide (476.00 pg/mL vs. 320.00 pg/mL, *p* = 0.007), creatine kinase (65.30 U/L vs. 54.60 U/L, *p* = 0.006), and lactate dehydrogenase (206.60 U/L vs. 198.45 U/L, *p* = 0.045). These patients also demonstrated significant cardiac structural and functional abnormalities, manifested as reduced left ventricular ejection fraction (*p* = 0.006), higher prevalence of cardiomegaly (86.0% vs. 71.3%, *p* = 0.006), and increased incidence of pleural effusion (46.7% vs. 24.0%, *p* < 0.001). These laboratory correlates validate dampness as a terminal‐stage marker of advanced ventricular remodeling and interstitial fluid overload [43, 44].

The geospatial stratification analysis of six regions revealed a climatic–pathophysiological gradient in syndrome clustering and progression, wherein conserved core elements (blood stasis, qi deficiency, phlegm) interact with region‐specific modifiers. This heterogeneity underscores the TCM principle of “correspondence between man and nature” (Tian Ren Xiang Ying), where environmental stressors shape the host's physiological response. Northern China's cold–dry climate manifested in yang deficiency–qi stagnation dominance and blood stasis→cold congealment transitions. Pathophysiologically, this aligns with the mechanism of cold‐induced sympathetic activation and vasoconstriction, which increases hemodynamic shear stress and promotes thrombotic events—the biological equivalent of “cold congealment.” This pattern is corroborated by nationwide epidemiological surveys conducted between 2012 and 2014 [10]. In contrast, humid subtropical regions, encompassing East, South Central, and Southwest China, exhibited accelerated phlegm–stasis synergy and qi‐yin deficiency cascades. Theoretically, persistent environmental humidity encumbers the spleen's transformation function, leading to the accumulation of pathological metabolites (phlegm) that obstruct vascular networks. These patterns aligned with clinical observations from Guangdong and Hainan provinces, where persistent environmental dampness exacerbated phlegm–turbidity constitutions, necessitating dampness‐targeted therapeutic strategies [45, 46]. Contemporary epidemiological evidence parallels these climate‐related findings. A study in Ethiopia demonstrated a significant positive association between average annual water vapor pressure (1970–2000) and the 10‐year CHD risk [47]. Similarly, a longitudinal trajectory analysis of CHD mortality across Turkey revealed that environmental factors—particularly air quality and climatic conditions (humidity, sunlight and temperature variations)—significantly correlated with regional CHD mortality [48]. Dietary patterns further modulated these gradients: Southwest China's spicy, greasy diets and South China's sweet, sticky foods correlated with spleen–stomach dysfunction and phlegm–turbidity predominance. Concurrently, coastal populations’ high‐protein diets (primarily fish and shrimp) synergized with humid climates to amplify phlegm–stasis pathogenesis [10, 49, 50]. These multidimensional interactions between climate, diet, and pathophysiology validated the TCM principle of “therapy tailored to locality” (San Yin Zhi Yi), underscoring the importance of geographically personalized CHD management protocols.

Shifting climatic patterns, pollution exposure, dietary habits, and lifestyle behaviors have also driven epoch‐dependent variations in CHD syndromic clustering and progression pathways across the 2000–2023 study period. Compared to 2000–2009, the subsequent decade witnessed cold congealment's decline as an independent node alongside disaggregated yang deficiency and qi stagnation domains. These syndromic shifts coincided with global warming trends that potentially attenuated cold pathogen dominance according to TCM theory. This temporal shift mirrored environmental epidemiological observations in Western populations. A German time‐stratified case‐crossover analysis comparing temperature‐related MI incidence between 1987–2000 and 2001–2014 found that cold exposure exclusively triggered MI in the earlier period (relative risk [RR] 1.26; 95% CI 1.08–1.47), while heat‐related MI risk significantly escalated in the latter epoch (RR 1.14; 95% CI 1.00–1.29) [51]. A meta‐analysis further quantified a 2.8% elevation in coronary heart disease risk per 1°C temperature rise above regional baselines [52], corroborating climate warming's cardiovascular impacts. Critically, air pollution synergized with rising temperatures to amplify CHD pathogenesis. A 2017 multi‐city European study demonstrated heightened pollution effects on cardiovascular mortality during high‐temperature days [53], while ozone‐related fatalities disproportionately increased under warmer conditions [54]. Although mechanistic clarity remains incomplete, this synergistic effect likely stemmed from compounded oxidative stress, systemic inflammation, and endothelial dysfunction, where pollutants and heat jointly impair nitric oxide bioavailability, accelerating atherosclerotic plaque instability and thrombotic propensity [55].

Critically, the post‐2020 CHD population exhibited a paradigm shift toward blood stasis–phlegm synergy and yin deficiency–blood stasis interactions, reflecting secular trends in lifestyle and diagnostic paradigms. A longitudinal analysis comparing syndromic distributions across four epochs from 1970 to 2021 revealed persistent increases in blood stasis and phlegm–turbidity prevalence from 1970–2010, with continued growth in 2010–2021. Concurrently, qi/yang/yin deficiency proportions declined while qi stagnation rates remained stable [56]. These shifts correlated with modern dietary transitions in China. National meat and dairy consumption doubled over three decades [57, 58], disproportionately elevating saturated fat intake and promoting lipid peroxidation. Theoretically, this validates the TCM principle that “the spleen constitutes the source of phlegm” (Pi Wei Sheng Tan Zhi Yuan). Modern high‐calorie diets impose a heavy metabolic load on the spleen (digestive system), impairing its transport and transformation functions. Consequently, accumulated pathological metabolites (phlegm) interact with vascular stagnation (stasis). This “phlegm–stasis” interaction likely serves as the macro‐syndromic manifestation of metabolic syndrome and chronic low‐grade inflammation driven by insulin resistance. However, regional surveys indicate suboptimal dietary balance among Chinese elderly populations, with only 12.1%–23.7% meeting recommended meat, vegetable, and fruit intake guidelines [59], potentially exacerbating metabolic dysregulation. Concurrently, sedentary behavior prevalence surged post‐2010, with 22.3% (95% CI 20.9% – 23.8%) of adults failing to achieve physical activity targets by 2018 [60]. From a TCM perspective, this reduced physical activity may impair yang qi circulation and accelerate stasis accumulation in susceptible individuals. While these factors explain syndromic shifts, diagnostic subjectivity must be acknowledged as a potential confounder. Evolving academic emphasis on phlegm–stasis syndromes, particularly post‐2010, may have inflated their reported prevalence due to heightened clinical vigilance. This potential diagnostic bias, superimposed on genuine pathophysiological changes, underscores the necessity for standardized syndromic criteria to differentiate true epidemiological trends from nosological shifts in the TCM literature.

Capturing the spatiotemporal fluidity of TCM syndromes requires moving beyond simple estimates of marginal prevalence. The real methodological challenge lies in simultaneously mapping how syndrome elements cluster structurally, how they reconfigure across varying contexts, and—crucially—deciphering the probabilistic directionality of their evolution along the disease course. While conventional meta‐analytic workflows excel at pooling prevalence data, they lack the architectural depth to expose network‐like dependencies or sequential progressions. Similarly, relying solely on repeated cross‐sectional snapshots often yields fragmented insights [10, 11], whereas mining electronic health records frequently hits a wall of unstructured noise and missing data that obscure clinically coherent progression modeling [61]. To bridge this gap, we engineered a hybrid, AI‐augmented framework capable of synthesizing vast literature corpora into dynamic progression models. By rigorously processing 57,971 publications through active learning prioritization and semantic validation, our pipeline achieved an unprecedented resolution in mapping syndrome transitions across 29 provinces and two decades—a scope effectively unattainable via conventional manual methods. Our approach moves beyond a monolithic analysis by integrating three distinct statistical layers: random‐effects meta‐analysis to establish robust baselines amidst heterogeneity; hierarchical clustering to delineate stable versus context‐dependent syndrome modules; and, most significantly, a tailored cSPADE algorithm designed to extract stage‐ordered transition rules.

In contrast to standard sequential mining tools such as Apriori and GSP, which often become computationally intractable at this scale [62], our optimized cSPADE implementation utilized depth‐first search and strict lift filtering (threshold >1.0). This ensured that we extracted only those transition rules where the association strength exceeded random chance, thereby prioritizing clinical salience over mere statistical frequency [63, 64]. The theoretical linchpin of this strategy is the principle of “pseudo‐longitudinal inference.” We posit that by ordering cross‐study evidence according to distinct disease stages, one can approximate population‐level trajectories. This assumes that baseline syndrome frequencies—rigorously harmonized via NLP dictionaries to minimize subjective noise—act as reliable proxies for dominant pathophysiological states, even if they represent probabilistic population patterns rather than deterministic individual courses. Ultimately, the resulting visualization of trajectories and modularity does more than just display data; it empirically grounds the classical “Treatment in Accordance with Three Categories of Etiologic Factors” doctrine in modern computation, offering a reproducible pathway to reconcile traditional wisdom with the rigor of large‐scale analytics [65].

Our study demonstrated literature mining's viability for modeling syndrome evolution across 29 provinces and two decades, achieving high temporal consistency with recently published observational studies. Nevertheless, several important limitations warrant discussion. First, the analysis was inherently dependent on the quality and methodological consistency of published TCM studies. Although standardized dictionaries and NLP techniques were employed for semantic alignment, TCM syndrome diagnosis involves a degree of subjectivity, and diagnostic criteria may vary across the included studies, leaving potential diagnostic heterogeneity as a residual confounding factor. Quality assessments identified a relatively high risk of bias in follow‐up outcome reporting among approximately half of the included case series. However, this methodological limitation does not compromise the validity of our core findings, as all analyses exclusively utilized baseline syndromic data obtained prior to therapeutic interventions. Second, spatiotemporal disparities in research infrastructure and data stratification resulted in uneven data coverage. Regionally, Southwest China contributed less than 2% of the included studies, potentially limiting the generalizability of findings in this region. Temporally, the 2020–2023 period spans only four years, whereas the preceding epochs each cover a decade; this variation in temporal granularity warrants cautious interpretation of the trend magnitude in the most recent period. Third, the predominance of mixed‐stage cohorts in our analysis (*n* = 374) constrained stage‐stratified sample sizes and precluded combined stage–region–period stratification. This limitation reflects the broader clinical research paradigm wherein CHD studies frequently aggregate disease stages without explicit stratification. Furthermore, our investigation identified substantial commonalities in syndrome distribution patterns across different disease stages, providing methodological justification for including all available literature in our geographic and temporal stratification analyses. Fourth, as an epidemiological investigation based on aggregated data, this study focuses strictly on the prevalence and distribution patterns of syndromes rather than therapeutic evaluation. Consequently, the current dataset lacks the direct prescription–outcome evidence required to validate clinical efficacy. Therefore, the observed syndrome patterns should be interpreted as macroscopic epidemiological references, which do not directly translate into specific therapeutic interventions or clinical decision‐making protocols without further patient‐level validation.

In summary, this large‐scale spatiotemporal analysis of 234,656 CHD patients delineates blood stasis, qi deficiency and phlegm as the conserved pathogenic anchors in Chinese populations, interacting dynamically with qi‐yin dual deficiency and phlegm–turbidity across disease progression stages. These syndrome patterns demonstrated significant geographic variation across China's diverse climatic regions and underwent substantial temporal shifts during the 2000–2023 period, reflecting environmental, dietary, and lifestyle changes. Our findings highlight the importance of developing geographically personalized and temporally adaptive TCM therapeutic strategies for CHD management, while underscoring the value of AI‐augmented literature mining as a viable approach for modeling syndrome evolution across large spatiotemporal scales.

## Funding

The study was jointly supported by the Natural Science Foundation of Beijing, China (7244507), National Natural Science Foundation of China (82405243, 82174532), China Association of Chinese Medicine's 2025–2027 Young Talent Support Project (2025–QNRC2–B07), China Academy of Chinese Medical Sciences key collaborative project of Innovation Fund (CI2022C004–01), and Outstanding Young Scientific and Technological Talents Training Program of the China Academy of Chinese Medical Sciences, funded by Basic Research Operating Expenses (ZZ19–YQ–033).

## Conflicts of Interest

The authors declare no conflicts of interest.

## Supporting information




**Figure S1**. All visualization of stage‐specific syndrome element clusters derived from agglomerative hierarchical clustering. Heatmaps encode co‐occurrence frequencies through color gradients (darker hues = higher similarity), with dendrograms illustrating cluster hierarchy. (A) Stable stage; (B) Active stage; (C) Post‐interventional stage; and (D) End stage.
**Figure S2**. All results from the cSPADE algorithm on different CHD stages with 0.1 and above support and confidence. (A) Stable stage; (B) Active stage; (C) Post‐interventional stage; and (D) End stage.
**Figure S3**. All six‐panel visualization of geographic‐region‐specific syndrome element clusters derived from agglomerative hierarchical clustering. Heatmaps encode co‐occurrence frequencies through color gradients (darker hues = higher similarity), with dendrograms illustrating cluster hierarchy. (A) North China; (B) Northeast China; (C) East China; (D) South central; (E) Southwest China; and (F) Northwest China.
**Figure S4**. All results from the cSPADE algorithm on different geographic‐region‐specific syndrome element clusters derived from agglomerative hierarchical clustering. Heatmaps encode co‐occurrence frequencies through color gradients (darker hues = higher similarity), with dendrograms illustrating cluster hierarchy. (A) North China; (B) Northeast China; (C) East China; (D) South central; (E) Southwest China; and (F) Northwest China.
**Figure S5**. All Three‐panel visualization of epoch‐specific syndrome element clusters derived from agglomerative hierarchical clustering. Heatmaps encode co‐occurrence frequencies through color gradients (darker hues = higher similarity), with dendrograms illustrating cluster hierarchy. (A) 2000–2009; (B) 2010–2019; and (C) 2020–2023.
**Figure S6**. All results from the cSPADE algorithm on different CHD stages with 0.1 and above support and confidence. (A) 2000–2009; (B) 2010–2019; and (C) 2020–2023.
