# Supplementary material for: AI‐Assisted Literature Mining Reveals Spatiotemporal Heterogeneity and Progression Trajectories of Traditional Chinese Medicine Syndromes in Coronary Heart Disease in China
Source: J Evid Based Med. 2026 Jul 7;19(3):e70162. doi: 10.1111/jebm.70162 (PMC13613246; doi:10.1111/jebm.70162)
Supplement: Supplementary file 1 [file JEBM-19-0-s001.docx]

**Supplementary materials**

**Supplementary Figure 1**. All visualization of stage-specific syndrome element clusters derived from agglomerative hierarchical clustering. Heatmaps encode co-occurrence frequencies through color gradients (darker hues = higher similarity), with dendrograms illustrating cluster hierarchy. (A) Stable stage; (B) Active stage; (C) Post-interventional stage; (D) End stage.

**Supplementary Figure 2**. All results from the cSPADE algorithm on different CHD stages with 0.1 and above support and confidence. (A) Stable stage; (B) Active stage; (C) Post-interventional stage; (D) End stage.

**Supplementary Figure 3.** All six-panel visualization of geographic-region-specific syndrome element clusters derived from agglomerative hierarchical clustering. Heatmaps encode co-occurrence frequencies through color gradients (darker hues = higher similarity), with dendrograms illustrating cluster hierarchy. (A) North China; (B) Northeast China; (C) East China; (D) South central; (E) Southwest China; (F) Northwest China

**Supplementary Figure 4.** All results from the cSPADE algorithm on different geographic-region-specific syndrome element clusters derived from agglomerative hierarchical clustering. Heatmaps encode co-occurrence frequencies through color gradients (darker hues = higher similarity), with dendrograms illustrating cluster hierarchy. (A) North China; (B) Northeast China; (C) East China; (D) South central; (E) Southwest China; (F) Northwest China.

**Supplementary Figure 5.** All Three-panel visualization of epoch-specific syndrome element clusters derived from agglomerative hierarchical clustering. Heatmaps encode co-occurrence frequencies through color gradients (darker hues = higher similarity), with dendrograms illustrating cluster hierarchy. (A) 2000-2009; (B) 2010-2019; (C) 2020-2023.

**Supplementary Figure 6**. All results from the cSPADE algorithm on different CHD stages with 0.1 and above support and confidence. (A) 2000-2009; (B) 2010-2019; (C) 2020-2023.
